# Überwachungskapitalistische Biopolitik: Big Tech und die Regierung der Körper

**DOI:** 10.1007/s41358-021-00309-9

**Published:** 2022-02-01

**Authors:** Felix Maschewski, Anna-Verena Nosthoff

**Affiliations:** 1 Institut für deutsche Literatur, Humboldt-Universität zu Berlin, Dorotheenstr. 24, 10117 Berlin, Deutschland; 2 Institut für Politikwissenschaft, Universität Wien, Universitätsstraße 7, 1010 Wien, Österreich

**Keywords:** Big Tech, Biopolitik, Überwachungskapitalismus, Pandemie, Infrastrukturelle Macht, Gesundheitswesen, Big Tech, Biopolitics, Surveillance capitalism, Pandemic, Infrastructural power, Health care

## Abstract

Der Artikel analysiert den Einzug Big Techs (der Fokus liegt auf Apple und Alphabet) in den Gesundheitsmarkt und beschreibt in Anlehnung an Michel Foucault und Shoshana Zuboff das Konzept einer „überwachungskapitalistischen Biopolitik“. Ziel ist, die Ausweitung des „Datenextraktivismus“ im Gesundheitswesen und der Gesundheitsforschung machtkritisch einzuordnen und damit einen Trend in der digitalen Gesundheitsfürsorge zu problematisieren, der sich in den letzten Jahren und, wie wir zeigen, besonders während der Coronakrise beschleunigt und ausgefächert hat. Anhand wissenschaftlicher und kommerzieller Projekte sowie Kooperationen im Bereich public health wird deutlich, dass zeitgenössische Formen der Biopolitik keineswegs auf staatliche Regime beschränkt sind. Stattdessen sind sie zunehmend über private Technologieunternehmen vermittelt, die dabei nicht nur intime Verhaltens- und Vitaldaten akkumulieren, sondern – qua proprietärer Algorithmen – auch den Zugang zu diesen kontrollieren und schließlich ihren Einfluss in exklusive Services und Produkte überführen. Ein besonderer Akzent des Artikels liegt zudem auf der voranschreitenden Verbreitung sogenannter Wearable-Technologien (Smartwatches etc.), über die sich nicht nur die herausgehobene Marktposition der Konzerne, sondern – in der Entwicklung von einem „quantifizierten Selbst“ zu einem „quantifizierten Kollektiv“ – auch ihre epistemische bzw. „infrastrukturelle Macht“ konkretisiert. Entgegen einer einseitig repressiven Perspektive auf biopolitische Praxen zeigen wir schließlich Ansätze einer Demokratisierung „überwachungskapitalistischer Biopolitik“ auf. Hierbei heben wir vier Topoi hervor, die von zentraler Bedeutung sind: Privatsphäre bzw. individuelle Souveränität, demokratische Deliberation, Pluralismus und epistemische Gleichheit.

## Abstract (en)

The article introduces the concept of “surveillance-capitalist biopolitics” to problematize the recent expansion of “data extractivism” in health care and health research. As we show, this trend has accelerated during the ongoing Covid pandemic and points to a normalization and institutionalization of self-tracking practices, which, drawing on the “quantified self”, points to the emergence of a “quantified collective”. Referring to Foucault and Zuboff, and by analyzing key examples of the leading “Big Tech” companies (e.g., Alphabet and Apple), we argue that contemporary forms of digital biopolitics are privatized, opaque, flexible, and not limited to the state. Instead, especially through the integration of wearable technologies, the biopolitical regulation of bodies is increasingly mediated by private tech companies. These companies rely on a questionable narrative of participation, responsibility, and care despite owning, and ultimately controlling, access to intimate health data and the proprietary algorithms mediating this data. The article shows that the proliferation of “surveillance-capitalist biopolitics” ultimately strengthens not only market power but also the epistemic and infrastructural power of the data-owning and gadget-producing firms. Finally, against an exclusively repressive and negative reading of biopolitics, and to effectively counter the forms of power emerging from surveillance-capitalist biopolitics, we propose four dimensions that are central to its democratization—namely *privacy/individual sovereignty, democratic deliberation, pluralism*, and *epistemic equality.*

„Es könnte sein,“ schreibt der französische Philosoph Gilles Deleuze Anfang der 1990er-Jahre in *Postskriptum über die Kontrollgesellschaften*, „daß alte Mittel, die den früheren Souveränitätsgesellschaften entlehnt sind, auf den Plan treten, wenn auch mit den nötigen Anpassungen. Entscheidend ist, daß wir am Beginn von etwas Neuem stehen.“ Deleuze beschreibt, ausgehend von Michel Foucaults Machtanalytik, „winzige Beispiele“ eines sukzessiven Regimewechsels – vom „*Gefängnis*-*Regime*“ bis zum „*Unternehmens-Regime*“ –, diagnostiziert eine „Krise der Institutionen“ und expliziert etwa für das „*Krankenhaus*-*Regime*: die neue Medizin „ohne Arzt und Kranken“, die potenzielle Kranke und Risiko-Gruppen erfaßt, was keineswegs von einem Fortschritt hin zur Individuierung zeugt, wie man sagt, sondern den individuellen oder numerischen Körper durch die Chiffre eines „dividuellen“ Kontroll-Materials ersetzt“.^1^ Diese gesellschaftsphilosophische Spekulation über den „fortschreitende[n] und gestreute[n] Aufbau einer neuen Herrschaftsform“ (1993, S. 261–262, Hervorh. i. Orig.), die sich mit der Einführung des World Wide Web und der invasiven Verbreitung kybernetischer Technologien (vgl. Virilio 1993) zu konturieren begann, scheint in der „digitalen Konstellation“ (Berg et al. 2020) der Gegenwart, die von ubiquitärer Vernetzung, der expansiven Nutzung großer Datenbanken, algorithmischer Risikoanalysen und smarter, sensorischer Erfassungsmechanismen geprägt wird, an Plastizität zu gewinnen.^2^

Im Anschluss an Deleuzes Überlegungen untersucht der Beitrag den Status quo des prognostizierten „Krankenhaus-Regimes“. Dabei wird anhand der programmatischen Verbreitung von „Kontrollformen mit freiheitlichem Aussehen“ (Deleuze 1993, S. 255) bzw. tragbaren Kleinstcomputern, sogenannter Wearables (vgl. Nosthoff und Maschewski 2019; Mück et al. 2020), eine Regierungsform dargestellt, die wir in Anlehnung an Michel Foucault und Shoshana Zuboff als *überwachungskapitalistische Biopolitik*^3^ beschreiben und in machtkritischer Perspektive plausibilisieren. Dafür wird zunächst verdeutlicht, wie sich die Großkonzerne von Big Tech^4^ – das Augenmerk liegt hier auf Alphabet (Google) und Apple – im Gesundheitssektor als Forschungsinfrastrukturen, Dienstleister und Produktentwickler etablieren, und wie ihre plattformökonomischen bzw. überwachungskapitalistischen Praxen über smarte Technologien (Smartwatches etc.) und explorative Datenanalysen Einzug in eine flexiblere, dezentralere Gesundheitsversorgung halten. Die überwachungskapitalistische Biopolitik bestimmt sich dabei als ein Set kybernetischer Kontroll- und kapitalistischer Marktmechanismen, das durch datafizierte Regulierungsmaßnahmen der Körper, d. h. durch die feedbacklogische Mediation korrelierter, biometrischer Idealwerte, beständig neue Anpassungsdynamiken der Individuen ermöglicht und erfordert. Diese Mechanismen entfalten, so die These, ihre Wirksamkeit weniger über feste, statische Vorschriften als über fluide, personalisierte Mitschriften. Sie bilden Milieus subtiler Korrekturen und „tragbarer Kontrolle“ (Maschewski und Nosthoff 2020) und erfassen mit den Verhaltens- und Vitalwerten nicht nur das Leben und die *Lebensweisen*, sie etablieren mit den Verfahren der Selbst- und Fremdüberwachung auch eine infrastrukturelle Macht, die (biopolitische) Standards setzt.^5^ In diesem Zusammenhang ergibt sich ferner eine plattformökonomische, epistemische Asymmetrie, die sich nicht allein in der Entwicklung exklusiver Produkte und Services materialisiert, sondern auch die Horizonte einer sowohl *privatisierten *wie auch* teilprivatisierten* Biopolitik im Modus von Public-private-Partnerships (z. B. die Kollaborationen Singapurs mit Fitbit und Apple) perspektiviert.

Der Beitrag knüpft an die Forschung zu Biopolitik unter digitalen Bedingungen an, aktualisiert und erweitert sie jedoch unter den Aspekten aktueller Machtkonstellationen und Produktionsdynamiken im Technologiesektor (vgl. Reichert 2018; Hille 2016; Cheney-Lippold 2011; Christiaens 2020).^6^ In der Folge liegt der Fokus weniger auf der bereits vielfach, u. a. in Gouvernementalitätsstudien analysierten Erfassung eines „quantifizierten Selbst“ (vgl. Whitson 2015; Neff und Nafus 2016; Bernard 2017; Hepworth 2019), als vielmehr auf der Hervorbringung eines „quantifizierten Kollektivs“ (vgl. Nosthoff und Maschewski 2019, S. 71), das neue medizinische Entwicklungen und gesundheitspolitische Potenziale, aber auch gesellschaftliche Verwerfungen zu produzieren vermag (vgl. Maturo und Moretti 2018).

In dieser Rahmung folgt die Argumentation durchaus einer „hyperbolischen“ Kritik, die sich im Sinne Foucaults besonders auf die „gefährdete Seite des Sozialen“ konzentriert (vgl. Saar 2007, S. 316). Dabei geraten vor allem Ambivalenzen in den Blick: So wird nicht nur analysiert, wie die überwachungskapitalistische Biopolitik Verheißungen der Partizipation mit kybernetischen Kontrolllogiken verbindet und dabei zentralisiert und dezentral, kollektivierend und individualisierend zugleich operiert.^7^ Deutlich wird auch, dass die Produkte und Projekte der Tech-Konzerne stets mit demokratischen Narrativen – zumeist annoncieren sie emanzipative Versprechen (neues Körperwissen, kollektive Praxen der Fürsorge, Demokratisierung der Forschung) – aufgeladen werden, dabei aber immer auf die Manifestation ökonomischer Herrschaftsstrukturen im Zeichen „proprietärer Märkte“ (Staab 2019, S. 174) zielen. Entgegen einer einseitig repressiven Perspektive auf biopolitische Praxen^8^ werden zum Schluss des Beitrags, kontextualisiert mit jüngeren Entwicklungen in der Covid-19-Pandemie, auch Ansätze einer Demokratisierung überwachungskapitalistischer Biopolitiken aufgezeigt. Hierbei heben wir vier Topoi hervor, die von zentraler Bedeutung sind:* (1.) Privatheit bzw. informationelle Selbstbestimmung, (2.) demokratische Deliberation, (3.) Pluralismus und (4.) epistemische Egalität*.

## Big Health: Dynamiken im Gesundheitsbereich

Dass Tech-Monopolisten, unter ihnen besonders Alphabet respektive Google, seit geraumer Zeit sämtliche Bereiche unseres Lebens kartografisch vermessen, ist spätestens seit Zuboffs (2018) einschlägiger Analyse zum „Überwachungskapitalismus“ en detail bekannt. Beispielhaft schildert die Wirtschaftswissenschaftlerin, wie die prominentesten Technologieunternehmen der westlichen Hemisphäre eine neue, digitale Spielart des Kapitalismus entwickelt haben, deren Wertschöpfungsmodell auf der Extraktion, Akkumulation und Analyse von Daten beruht. Dabei werden die (Verhaltens‑)Daten bzw. die „Verhaltensüberschüsse“, die beim Gebrauch digitaler Produkte und Services (z. B. beim Liken bei Facebook) von den Nutzer:innen selbst produziert werden, erfasst, abgeschöpft und von den Unternehmen, algorithmisch gestützt, in „Vorhersageprodukte“ (Zuboff 2018, S. 22) überführt, die sich ihrerseits gerade in der Werbebranche gewinnbringend verkaufen und verwerten lassen. Zuboff bestimmt das Ziel dieser Operationen in der ökonomischen Orchestrierung zukünftiger Entscheidungs- und Verhaltensweisen, gar in ihrer Modifikation, und attestiert dem Überwachungskapitalismus in Anlehnung an Hannah Arendts *Elemente und Ursprünge totaler Herrschaft* eine gesellschaftliche Dominanz, die demokratieschädlich verfährt (vgl. Zuboff 2018, S. 590f.).

In den vergangenen Jahren haben die Tech-Monopolisten die überwachungskapitalistischen Strategien der Datenextraktion auch im Bereich des Digital Health – insbesondere vermittelt über Wearables – intensiviert und ausgeweitet. Diese Entwicklungen sind von der Forschungsliteratur bislang allenfalls randständig in den Blick genommen worden,^9^ und so blieb auch im Kontext der Untersuchungen zum plattformökonomischen bzw. digitalkapitalistischen Komplex (vgl. Srnicek 2018, Staab 2019) analytisch unterbelichtet, dass der Großteil der Tech-Elite in der Kartierung der Körper bzw. dem „beständige[n] und unmerkliche[n] Eindringen von Sendekanälen in das gesellschaftliche „Fleisch““ (Lyotard 2007, S. 254) selbst ein lukratives Feld für Forschung und Entwicklung erkannt hat – und dieses, wie besonders in der Covid19-Pandemie deutlich wird, immer konzentrierter erschließt (vgl. Cosgrove et al. 2020, Maschewski und Nosthoff 2021a). In der Tat lässt sich eine Expansionsdynamik erkennen, in der sich die Konzerne von Big Tech über die Etablierung ihrer tragbaren Devices, ihrer Health-Apps, KI-Systeme oder Cloud-Services und die damit verbundene beständige Akkumulation von Verhaltens- und Gesundheitsdaten immer mehr zu dem entwickeln, was man als *Big Health* bezeichnen kann.^10^ Verwunderlich ist diese Ambition kaum, soll doch allein das Volumen des digitalen Gesundheitsmarktes bis 2025 979 Mrd. Dollar annehmen (vgl. Neumann et al. 2020).

Vor diesem Hintergrund werden im Folgenden aktuelle Projekte von Apple und Alphabet (Google) genauer beleuchtet, die den biometrischen Datenextraktivismus nachvollziehbar machen und aufzeigen, wie die Tech-Elite – nicht selten in Kooperation mit etablierten, medizinischen Forschungseinrichtungen – die multisensorische, ubiquitäre Vernetzung in immer umfänglichere Services und Produkte überführt. Die anschließende Analyse arbeitet heraus, wie sich plattformökonomische bzw. überwachungskapitalistische (aber auch staatliche) Praxen und Geschäftsmodelle erweitern, vertiefen und biopolitisch wirksame Souveränitäten akzentuieren.

## Apple: Forschung und Versicherung

Die Aktivitäten der Tech-Konzerne verdichten sich in einer prägnanten Aussage des Apple-CEOs Tim Cook, der bereits im Januar 2019 für Big Tech fast stellvertretend exemplifizierte: „[W]enn man einst […] die Frage stellte, was der größte Beitrag Apples für die Menschheit“ gewesen sei, werde es nur eine Antwort geben, nämlich „die Gesundheit“ (zit. N. Gurdus 2019; Ü. d. A.). Cooks Ambitionen richten sich besonders auf ein Endgerät, das zu einer signifikanten Schnittstelle für ein digitales Gesundheitsmanagement geworden ist: die *Apple Watch*. Denn war die Smartwatch noch bei ihrer Einführung 2015 kaum mehr als ein Accessoire, das dem Self-Tracker im Zusammenhang mit neoliberalen Körperidealen (vgl. Hakim 2015) über „die Feinsteuerung der Lebensweisen“ (Mau 2017, S. 181) zu mehr Aktivität, Leistung und Fitness verhelfen sollte, wurden zuletzt vor allem ihre Health-Funktionen konsequent ausgeweitet. So soll die Smartwatch nicht mehr nur den Umsatz täglicher Bewegungen berechnen oder die Selbstoptimierer:innen mit den sogenannten „Taps“, d. h. vibrierenden Benachrichtigungen, an bestimmte Zielwerte erinnern. Sie wird heute vor allem als „ultimative[s] Tool für ein gesundes Leben“^11^ beworben: Über die Verfeinerung der Sensorik werden Körperwerte wie die Herzfrequenz und der Sauerstoffgehalt im Blut, aber auch Biomarker (messbare Indikatoren für Krankheiten) getrackt. Damit bestimmt das Wearable sukzessive eine medizinische Diagnosefähigkeit (vgl. Drexler et al. 2020), die die Konzentration von der Erfassung der Fitness auf die Analyse der Gesundheit verschiebt.

Diese Dynamik geht mit einem Beleuchtungswechsel einher. Denn der Apple-Konzern öffnete über die expansive Verbreitung der eigenen Devices zuletzt den Bezugsrahmen und entwickelt mit dem Ziel, die „Betreuung […] effizienter und persönlicher“ bzw. „menschlicher“ zu machen, für Krankenhäuser Technologien (Apps etc.), die helfen, „effektiv zu arbeiten“ bzw. „aus der Distanz mit Patienten in Verbindung zu bleiben.“ Dem iPhone, aber besonders der Apple Watch, kommen dabei Bedeutung zu, da sie neben dem Versprechen einer besseren und schnellen Zirkulation der Informationen auch über Schnittstellen, wie das „Research-“ oder „CareKit“ verfügen, die eine Teilung bzw. Weitergabe von Gesundheitsdaten und damit, wie es heißt, „bahnbrechende medizinische Forschung“^12^ ermöglichen. So wird qua Apple Watch nicht mehr allein ein „quantifiziertes Selbst“ (vgl. zu seiner Genealogie und Bedeutung Wolf 2010; Lupton 2016, Mämecke 2021) angesprochen, sondern ein „quantifiziertes Kollektiv“ (Nosthoff und Maschewski 2019, S. 71), d. h. es werden größere Nutzer:innen-Kohorten adressiert, die nun helfen sollen, auch die „medizinische[] Forschung zu demokratisieren, indem Kunden von Apple durch Nutzung ihnen vertrauter Technologien die Möglichkeit haben, an Forschungen teilzunehmen“.^13^ Eben dafür arbeitet Apple mit einer Reihe von medizinischen Instituten, Krankenhäusern und Universitäten zusammen, bildet Forschungsallianzen und über die aggregierten Datensätze sukzessive einen umfangreichen „kollektiven Referenzkörper“ (Mau 2017, S. 176ff.) aus, der kontinuierlich erweitert wird und neben neuen wissenschaftlichen Erkenntnissen auch die Einführung und Weiterentwicklung innovativer Produkte ermöglichen soll.^14^ Besonders deutlich wurde diese Konzeption erstmals in der von Stanford Medicine zwischen 2017 und 2018 durchgeführten „Apple Heart Study“, bei der die Wearable-gestützte Detektion von unentdecktem Vorhofflimmern erforscht wurde. Die Studie wies über die Daten von mehr als 400.000 Teilnehmenden nach, dass die Sensoren des Wearables Anomalien in der Herzfrequenz aufspüren können (vgl. Perez et al. 2019), und bildete den Ausgangspunkt einer Dynamik, die qua Big Data und algorithmischer Musteranalyse personenbezogenes Profiling mit kollektiver Gesundheitsfürsorge verbindet.

Diesem Kurs folgt der Konzern seither zielstrebig. Bereits 2019 wurde Apple Health Records, eine Art medizinische Gesundheitsakte, und bei der Präsentation der Apple Watch Series 5 auch die Apple „Research App“ vorgestellt, über die User an weiteren Forschungsprojekten partizipieren können. In Zusammenarbeit u. a. mit der World Health Organization und der Harvard School of Public Health untersucht man seither die Hörgesundheit („Apple Hearing Study“), den weiblichen Zyklus („Apple Women’s Health Study“) oder das Zusammenspiel von Bewegung und Herzgesundheit („Apple Heart and Movement Study“). Apple entwickelt seine Smartwatch damit zu einer grundlegenden Forschungsinfrastruktur, bietet man doch die notwendige Sensorik und explorativen Algorithmen für die Erhebung und Analyse vormals unzugänglicher Daten.

Neben der wissenschaftlichen Forschung erfährt das quantifizierte Kollektiv auch in plattformökonomischen Geschäftsmodellen eine neue Tragweite, bei denen besonders prämienbasierte Modelle von Krankenversicherungen in den Fokus treten.^15^ Gerade Apple hat sich in diesem Feld zuletzt sowohl als Hardwareproduzent als auch als Software-Entwickler profiliert und in Kooperation mit der Aetna-Versicherung eine Plattform entworfen, die Versicherte über die 2019 veröffentlichte App *Attain* individuell zugeschnitten zu einem gesünderen Lebensstil motivieren soll.^16^ Über die App können die Nutzer:innen der Apple Watch ihre Aktivitäten protokollieren, individuelle Zielwerte festlegen und auf Basis ihrer Verhaltens- und Vitaldaten Beratung für eine gesündere Lebensweise einholen. Da smarte Versicherungen zumeist auf Bonusmodelle, das gamifizierte Nudging (vgl. Tanninen et al. 2020; genereller: Sunstein und Thaler 2007) und programmatische Anreize setzen, werden die Versicherten zum Sammeln von Punkten angehalten. Wer dann an seiner Gesundheit arbeitet und seine Werte qua Device überwachen lässt, erhält feedbacklogisch Anerkennung und wird – klassisch behavioristisch – belohnt, indem sich die Apple Watch über erreichte Gesundheitsziele (von den erfüllte Trainingseinhalten bis zur verbesserten Fettverbrennung) abbezahlen oder auch ein Produkt von lizensierten Partnern (Amazon, Bestbuy, CVS (Aetna gehört zur Unternehmensgruppe), Nike oder Starbucks) erwerben lässt. Apple und Aetna bauen so über die Smartwatch eine Plattform für anreizinduziertes Gesundheitstracking auf, die Kund:innen datenbasiert mit persuasiven „Taps“ und attraktiven Angeboten von einer gesünderen Lebensführung überzeugen soll (vgl. Fogg 2002).^17^ „Überwachung ist“, wie der Soziologe Nils Zurawski für die digitalen Dienste von Apple und Co. ganz generell bemerkt, auch hier „kein feindlicher Akt des Misstrauens […], sondern ein im Akt des Konsums angelegter Service, also ein Feature“ (Zurawski 2021, S. 92).

Auf dem Markt der Lebensversicherungen hat sich die Wearable-gestützte Gesundheitsanalyse in den USA weiter differenziert und verschärft. So ist das Tragen eines Fitnesstrackers beim Lebensversicherer John Hancock für Neuversicherte nicht optional, es ist sogar obligatorisch, wobei neben der Apple Watch auch Googles Fitbit und das Amazon Halo angeboten werden.^18^ Im Rahmen der hauseigenen Vitality-Programme, die zu einer „more meaningful, engaging and holistic experience“^19^ führen sollen, werden bei John Hancock körperliche Aktivität (10.000 Schritte sind hier das Maß der Wahl) und das tägliche Konsum- und Ernährungsverhalten in einem zentralen Gesundheitsscore erfasst, der dann die Höhe der individuellen Versicherungsgebühren bestimmt – die sogenannten *pay as you live*-Tarife. Die versichernde Leistungsschau bzw. ständige Fernüberwachung dient dann nicht mehr einer wissenschaftlichen Erkenntnis. Die smarte Versicherung will auf der kollektiven Vermessungsgrundlage direkt auf das Verhalten – man nennt dies „behavioral underwriting“ (vgl. Zuboff 2018, S. 248ff.) – der Kund:innen einwirken, es optimieren bzw. über eine enganliegende, gamifizierte Umwelt aus Services und Feedbacks, d. h. „Verhaltensmodifikationsmitteln“ (Zuboff 2018, S. 23) neu motivieren (Sullivan 2018). Fast folgerichtig erscheint es dann, dass die Versicherung auch über spezifische, algorithmisch orchestrierte Interventionsmechanismen verfügt, die sich im Modus des verhaltensökonomischen Nudgings scheinbar stark von klassischen Disziplinarmaßnahmen unterscheiden. Dennoch demonstrieren sie eine privatisierte „Biopolitik des Marktes“ (Mau 2017, S. 119), die über individuelle Reproduktionsweisen informiert und das Verhalten über Schubser in die „richtige Richtung“ (Sunstein und Thaler 2007, S. 15) zu strukturieren, zu normieren sucht. Konkret heißt dies etwa bei John Hancock: Erreichen die Versicherten über zwei Jahre ihre Aktivitätsziele, behalten sie die Apple Watch ohne Zuzahlung, können an Bonusprogrammen teilnehmen und ihre Versicherungsbeiträge um bis zu 25 % senken. Verfehlen sie die Benchmarks, muss die Smartwatch nachträglich abbezahlt werden. Die Klient:innen bleiben von allen Prämien und Rabatten ausgeschlossen. Die Effizienz dieser Modelle wurde empirisch untersucht: Mit der Apple-Watch ausgerüstete Versicherte steigerten ihre Aktivität pro Monat um durchschnittlich 34 % (vgl. Hafner et al. 2018). Damit markieren das beständige Scoring, Monitoring und die verhaltensbasierten Tarife für den Versicherer enorme Potenziale,^20^ da sie den Service der Gesundheitsförderung mit dem Versprechen der Kostensenkung (auch die der Versicherung) und einer genaueren Risikokalkulation verbinden.

Dieser Absicht folgen auch die Modi einer sich weiter ausprägenden Datenverknüpfung. So treten bei John Hancock die Gesundheitsdaten nicht nur ganz programmatisch mit Bewegungsprofilen, Konsum- und Ernährungsgewohnheiten der Versicherten in (Kor-)Relation. Über explorative Datenanalysen lassen sich auch in der Versicherten-Kohorte neue Vergleichskontexte generieren, die neben der Privilegierung bestimmter Verhaltensweisen und Lebensstile (bzw. Einkommensklassen) und der Sanktionierung von Abweichungen auch Statusbeziehungen organisieren und hierarchisieren. Soziale Ungleichheiten oder diskriminierende Praxen können im quantifizierten Kollektiv so durchaus fortgeschrieben, gar verstärkt werden (vgl. Maturo et al. 2018; explizit für John Hancock: vgl. Hilton 2021) und gerade die Personalisierung von Versicherungstarifen produziert nicht selten Effekte der Entsolidarisierung (vgl. Deutscher Ethikrat 2017, S. 234f.; Ellerich-Groppe 2021).^21^

Darüber hinaus werden auf „Verhaltensterminkontraktmärkten“ (Zuboff 2018, S. 251) die körperbezogenen Informationen nicht selten von externen Datenhändlern zweit- und drittverwertet (vgl. Lupton 2016, S. 119) und mit anderen Datenquellen verbunden: „This new practice“, erläutert Deborah Lupton die soziopolitischen Konsequenzen, „can affect people’s access to healthcare, credit, insurance, social security, educational institutions and employment options and render them vulnerable to unfair targeting by policing and security agencies“ (2016, S. 119). Damit intensivieren smarte Versicherungen im Zusammenspiel mit anderen intransparenten Instanzen besonders dann einen „polizeiliche[n] Blick“ (vgl. Bernard 2017, S. 103) auf den eigenen Körper, wenn die Existenz (wie bei ökonomisch und strukturell benachteiligten Personen) ohnehin schon prekär ist. Als Konsequenz wird ein asymmetrischer Anpassungsdruck im Sicherheitsdispositiv adressiert, der gerade in der privatisierten Form der Biopolitik eine „politics of *differential vulnerability“ *(Lorenzini 2021; Hervorh. i. Orig.) lesbar macht, die Verletzlichkeit in unterschiedlichem Maße ausstellt und ausbeutet, potenziert.

## Google: Gesundheitsmapping und neue Referenzen

Neben Apple bemüht sich besonders Alphabet sowohl um die Entwicklung avancierter Wearable-Technologien als auch um die wissenschaftliche Erfassung bzw. Kartierung der menschlichen Gesundheit. Eine kurze Analyse ausgewählter Projekte des Monopolisten ist schon deshalb gewinnbringend, weil sie beispielhaft aufzeigen, welche Regierungspraktiken – privatisierte wie teil-privatisierte – sich aus der überwachungskapitalistischen Expansion von Wearables ergeben.

Alphabets Bestrebungen im Gesundheitsmarkt haben sich bereits länger angekündigt, jedoch in den vergangenen drei Jahren im Zeichen von Big Health deutlicher nuanciert. So forschte der Konzern bereits an smarten Kontaktlinsen und OP-Robotern, investiert unablässig sowohl in Start-ups^22^ als auch in etablierte Firmen wie den Wearable-Hersteller Fitbit. Zuletzt konzentrierte man sich mit Google’s Health Division und dem Subunternehmen DeepMind vor allem auf KI-Anwendungen, die Krankheiten oder Krankheitsverläufe erkennen bzw. vorhersagen und damit ein besseres Gesundheitsmanagement in Kliniken ermöglichen sollen (vgl. Tomašev et al. 2019). Gerade in den Bereichen einer automatisierten, prädiktiven Diagnostik wurden nachhaltige Marktpotentiale erkannt, die allerdings auf die Akkumulation einer großen Menge von Patient:innendaten angewiesen sind. Aus diesem Grund ging Alphabet in den letzten Jahren Kooperationen mit externen Gesundheitsdienstleistern ein, über die man – häufig ohne das Wissen der Patient:innen – Millionen von Datensätzen über Krankheitsverläufe erwarb (vgl. Hurtz 2019). Zugleich wurden Projekte zur Datenakkumulation initiiert, die vorrangig auf Wearables setzen. Das Subunternehmen Verily, vormals Google Life Sciences, fungiert dabei als Spezialist, der immer wieder Großstudien ausrollt.

So arbeitet Verily im „Project Baseline“ zusammen mit Google, der Stanford und Duke University an Studien, die mal einzelne Krankheiten wie Typ-2-Diabetes oder Depressionen (z. B. in der Mood Study) erforschen, mal die Lebensweisen ganzer Alterskohorten vermessen. In der Health Study – eines der Kernprojekte – werden dafür seit 2018 10.000 Menschen über vier Jahre hinweg mit sogenannten Study Watches ausgestattet, um sämtliche Vitalwerte (bspw. Schrittzahl, Herzfrequenz, Schlafqualität) zu tracken. Zugleich sollen die Proband:innen kontinuierlich Fragebögen ausfüllen, Checkups in Kliniken und Tests – vom Seh- über den Blut bis zum Gentest – absolvieren, die dem Konzern in alle Bereiche des Lebens (und Sterbens), in die biologischen bzw. biorhythmische Höhen und Tiefen Einblicke eröffnen, und so eine ganz neue Form des „Reality Minings“ (vgl. Pentland 2015) ermöglichen. Alles unter dem Motto: „We have mapped the world. Now let’s map human health.“^23^

Im „Project Baseline“ erfassen Google und Verily so umfassend wie invasiv Gesundheits- oder Krankheitszustände, ihre Entwicklung, Bedingungen und Verläufe, um mehr über das Zusammenspiel von Körper und Geist, Individuum und Umwelt zu erfahren und die eigene Datenbasis permanent anzureichern. Das Projekt – „Baseline“ bedeutet wörtlich „Referenzwert“ oder „Vergleichslinie“ – zielt dabei auf die Konkretisierung und Differenzierung von kritischen Schwellenwerten, d. h. eine genauere Profilierung des Einzelnen im quantifizierten Kollektiv. Dabei setzt die „Health Study“ auf datenbasierte Gruppenbildung bzw. *Biocluster*, die über algorithmische Musteranalysen und Korrelationen erzeugt werden: Individuelle Vitalwerte müssen so nicht mehr allein mit bloßen Durchschnittswerten abgeglichen werden. Sie werden vielmehr ins Verhältnis zu anderen, analysierten Profilen gebracht, d. h. Menschen mit ähnlicher Merkmalsausprägung (vom Alter, den Körpermaßen oder Lebensstilen bis hin zu spezifischen Symptomen) bzw. „people like you“. Mit fast panoptischer Ein- und Übersicht sollen die Werte dabei in Feedbackschleifen zwischen Patient:innen und Forscher:innen besprochen, abgeglichen und angepasst werden. Diese Wissens‑, Kontroll- und Vergleichslandschaften nähren das Versprechen einer immer effizienteren Kartierung im Zeichen der Gesundheitsprävention, die in diesem Fall von dem Konzern selbst besorgt wird.

Als Medien der Präventionsmaßnahmen fungieren algorithmisch generierte und aus der Analyse der Datensätze ermittelte Metriken, deren Erstellung auch das Ziel (bzw. den Namen) des Projekts selbst beschreibt. So gilt es, wie es bei Google und Verily heißt, die grundlegende „Baseline“ des gesellschaftlichen Körpers zu bestimmen, d. h. „a well-defined reference […] of good health“ zu *formulieren*. Dafür sammele man umfassende Gesundheitsdaten, „to better understand the transition from health to disease and identify additional risk factors for disease.“^24^ Aus den metrischen Kurven sollen letztlich verbindliche Referenzwerte, aus der datenbasierten Empirie orientierende Normalitäten destilliert werden, die für jedes Biocluster überindividuelle Gültigkeit versprechen. Mindestens will man von dem Ist- auf die Sollwerte der Gesundheit schließen, um die nötige Anpassungsleistung der Individuen zu definieren, d. h. die Horizonte aufzeigen, wie sich die Regulierung von Körper und Geist via smart Device situativ flexibel, mathematisch effizient, in Echtzeit und durchaus eigendynamisch bewerkstelligen lässt. Klar wird in dem Projekt, dass es sich bei den Wearables mehr als nur um „Lifestyle-Produkte handelt, die nicht darauf ausgelegt sind, medizinische Diagnosen im engeren Sinn zu stellen“ (Mau 2017, S. 116). Vielmehr werden die digitalen Selbsttechnologien gerade dahingehend perfektioniert, kategorische Distinktionen zu bestimmen, Diagnosen zu stellen und übergeordnete Urteile zu fällen, „to better characterize health“.^25^

Obgleich die Teilnehmenden im „Project Baseline“ Leib und Leben unter eine ständige, externe Kontrolle stellen,^26^ verspricht der Konzern jedem Einzelnen, zum „partner“, zum Teil einer „community“ zu werden, die über das „redesign of the future of health“ nichts weniger als den „course of humanity“ verändert. In der konzerninduzierten „Sorgearbeit“ hat sich die Tonlage verschoben: Denn im Interesse an der Erstellung einer „comprehensive map of health that can one day better predict and prevent disease“ spiegelt sich kaum mehr die lokal-zentrierte Rationalität eines „quantifizierten Selbst“. Es geht stattdessen um kollektives Empowerment – „everyone should be able to participate“ –, verbunden mit dem altruistischen Aufruf, seine Vitaldaten vermittelt von Google und Co. zu re-sozialisieren bzw. der „community“ selbst zu überantworten. Jedes mit dem Wearable aufgezeichnete Datum beglaubigt in dieser Narration dann mehr als nur die eigene, singuläre Existenz. Eingespeist in hehre Ziele – „together, we can invent the future of data-powered healthcare“ – sollen sich auch die Bedeutungshorizonte einer datafizierten Solidarität eröffnen: „make your mark on the map of human health.“^27^

Mit dieser sozialen Rhetorik lässt Verilys „Project Baseline“ eine Gouvernementalität erahnen, die immer stärker auf die Körper der zu regierenden Subjekte konzentriert ist. Dabei etabliert sich eine epistemische und durchaus normative Autorität, die jüngst im Zuge staatlicher Programme und Public-private-Partnerships weiterentwickelt wurde.

## Die Regierung der Körper im Public-private-Partnership

Was anhand Apples und Alphabets bzw. Googles Ambitionen anschaulich wird, ist nicht allein der Einstieg zweier Tech-Monopolisten in den Gesundheitsmarkt. Vielmehr offenbart sich immer häufiger ein System- und Perspektivwechsel, der über die Vernetzung der individuellen auf einen Gesellschaftskörper zielt, damit auf ein soziales Gefüge oder ganz explizit auf die Bevölkerung als Ganze. Wenig verwunderlich ist dann, dass auch das „Health Promotion Board“ (HPB) der Regierung Singapurs im Verlauf der staatlichen „Smart Nation Initiative“ zuletzt zwei Kooperationen – mit Googles Fitbit („Live Healthy SG“) und der Apple Watch („LumiHealth“) – verkündete, die landesweit Wearable-basierte Gesundheitsprogramme anbieten. Ziel sei es, die Gesundheit der Bürger:innen qua Wearable zu analysieren, um diese über gamifizierte Challenges und finanzielle Vergütungen (bei „LumiHealth“ bis zu 380 Singapur-Dollar) und kommerzielle Belohnungen zu einem gesünderen Lebensstil zu animieren.^28^ „Innerhalb der App“, so heißt es etwa bei Apple, „begeben sich die Anwender mit einem freundlichen intergalaktischen Forscher auf die Reise durch unterschiedliche Welten, der sie durch Aufgaben führt, die auf Alter, Geschlecht und Gewicht abgestimmt sind. Dazu gehören wöchentliche Aktivitätsziele, die nicht nur durch Gehen, sondern auch durch Schwimmen, Yoga und andere Aktivitäten erreicht werden können. LumiHealth erinnert die Nutzer auch daran, Gesundheitsscreenings und Impfungen durchführen zu lassen. Darüber hinaus motiviert die App an Herausforderungen teilzunehmen, die darauf abzielen, Schlafgewohnheiten und Achtsamkeit zu verbessern und fördert eine bessere Auswahl an Lebensmitteln.“^29^

Obgleich in beiden Programmen die individuellen Daten mit dem HPB geteilt und in staatlich kontrollierten Datenbanken gespeichert werden; die Teilnehmenden kontinuierlich und je nach (Vital‑)Datensatz „personalized health advice and nudges“ zur Verhaltensoptimierung bzw. zum „behavior change“ erhalten, und so durchaus Modi der Fernüberwachung virulent werden, sollen die Programme, wie es im Zuge der Fitbit-Kooperation heißt, vor allem der Möglichkeit dienen, „that they [the Singaporeans, Anm. von d. Verf.] can take control of their own health.“^30^ Selbstermächtigung und Überwachung erscheinen hier fast ununterscheidbar, sodass die „Sorge um sich“ (Foucault) mit der Sorge um die Gesellschaft informationstechnisch rückgekoppelt, die quantifizierte Einzelne im Zusammenspiel von überwachungskapitalistischen Direktiven und fast schon staatskybernetischen Kontrolllogiken immer konsequenter in ein „metrisches Wir“ (Mau 2017) überführt wird. So fassen die Kooperationen nicht nur die Ambitionen von Apple und Google, ihre Produkte und Services überblickshaft zusammen. In ihnen zeichnen sich auch die Horizonte einer gouvernementalen Praxis ab, die Überwachung als konsumierbaren Service darstellt, die Individuen beständig analysiert und informiert, und so Leben und Lebensweisen reguliert bzw. regiert. Oder, wie es auf der Webseite des Apple Projekts geschrieben steht: „LumiHealth uses technology and behavioural insights to offer a unique health and wellness experience designed to delight, motivate, and reward Singaporeans and residents.“^31^

In dieser Perspektivierung bestimmen die Public-private-Partnerships die eingesetzten Wearables fast idealtypisch als Medien einer *überwachungskapitalistischen Biopolitik*. Sie markieren eine Regierungsform, die in der Folge als Erweiterung zu Foucaults Analysen der Regierung der Körper genauer akzentuiert wird.

## Überwachungskapitalistische Biopolitik

Versteht man die Mechanismen der Macht in Foucaultscher Lesart (2005, S. 256) als ein „Ensemble aus Handlungen, die sich auf mögliches Handeln richten“, als eine Konstellation, die „Anreize [bietet], verleitet, verführt“, etabliert sich mit der Wearable-basierten Infrastruktur, die die menschlichen Verhaltens- und Vitaldaten in Datenbanken erfasst und bewirtschaftet, ein eindringlicher, kybernetisch orchestrierter Modus der Biopolitik. Die im Zuge der neuzeitlichen politischen Ökonomie entstehende „Regierung der Bevölkerung“, die mit der „Geburt völlig neuer Taktiken und Techniken“ (Foucault 2006, S. 158f.) und dem „Eintritt des Lebens und seiner Mechanismen in den Bereich der bewußten Kalküle“ (Foucault 1977, S. 170) einherging, übersetzt sich in eine weitaus flexiblere, hautnahe Form. Tatsächlich wird in der neuen Spielart der datenextraktivistischen Erfassung und Aktivierung ein Regierungshandeln kenntlich. Wearables werden hier ganz im Sinne der biopolitischen Ausweitung eines möglichst lückenlosen „Regierungswissens“ (Foucault 2006, S. 159) eingebunden (vgl. Whitson 2015). Zugleich beschränkt sich ihr Gebrauch kaum mehr auf die „Biopolitik des Marktes“ (Mau 2017, S. 119), als die sich noch die (Selbst‑)Regulierung der Versicherungsprogramme beschreiben ließ. Die personalisierte Echtzeiterhebung erhält nun auch eine staatliche Steuerungsdimension (siehe „LumiHealth“), in der die Tech-Konzerne gleichwohl ihren Handlungsrahmen – Big Tech wird um die Facette des Big Health ergänzt – sukzessive ausbauen.^32^

Die Regierungskunst qua Wearable konzentriert sich so nach wie vor auf den Körper der Bevölkerung und bezieht sich, so formuliert es Maria Muhle (2013, S. 248) für Foucaults Begriff der Biopolitik, „in der Weise auf das Leben, dass sie ihm die Mittel zur Selbstregulierung bereit stellt“. Doch stehen mit den dezentraleren, feedbacklogischeren und partizipativeren „Technologien des Selbst“ (Foucault) Apparate zur Verfügung, die zwar oberflächlich einsehbare Daten – von Aktivitätskurven bis zum Zyklusstatus – produzieren, aber als Maschinen selbst Black Boxes bleiben (vgl. Maschewski und Nosthoff 2020). Jenseits der kausalen und statistischen Grundierung klassischer Biopolitik arbeiten die smarten Devices und ihre Services mit fluiden Datensets und proprietären Algorithmen, bei denen weder die Vergleichs- oder Bezugssysteme, Musteranalysen oder Korrelationen innerhalb der Datensätze noch die Programmatik des Quellcodes transparent sind. So wird die Ausrichtung der Körper durchaus selbstbestimmt, aktiv und eigeninitiativ von den Individuen vorgenommen. Aber die algorithmischen Verfahren der personalisierten Bewertung und Analyse implementieren immer auch ein ungleiches Sichtbarkeitsverhältnis, das konstitutiv auf Opazität gründet.^33^

Bereits die klassische, staatlich grundierte Bio-Macht richtete mit ihren periodischen Statistiken und zyklisch ermittelten Durchschnittswerten „die Subjekte an der Norm aus, indem sie sie um diese herum anordnet“ (Foucault 2005, S. 162). Doch bestimmen in der überwachungskapitalistischen Biopolitik vor allem die privaten Hersteller und Betreiber der Infrastrukturen, der Devices und Apps, den präjudizierenden, normierenden Rahmen^34^ – damit auch die Definitionen dessen, was Abweichung von der Norm bedeutet. So konturieren die Konzerne über das Gehäuse der schwarzen Kästen hinaus eine proprietär-datafizierte „Benennungsmacht“ (Bourdieu 1985; vgl. auch Mau 2017, S. 185ff.), über die sie eine epistemische Autorität zu untermauern und stets auszubauen suchen.^35^ Mit der Etablierung dieser neuen Mechanismen der Selbst- und Fremdüberwachung systematisieren sie eine Erfassungslogik, die mit einer „demokratischen Biopolitik“ (vgl. Schubert 2020a, b; Sotiris 2020; zu einer „affirmative biopolitics of care“ vgl. Christiaens 2022) konfligiert: Ihre Verfahren und Normativitäten sind seitens der Nutzer:innen weder verhandelbar noch sind sie deliberativ in repräsentativ-demokratischen Institutionen eingebettet.

Parallel zu diesen Entwicklungen folgen die Projekte stets der Prämisse, dass sich weniger durch Repression, als durch verlockende Kommunikation; weniger durch Verfahren der Disziplinierung, als durch kybernetisch orchestrierte Selbstregulierung sehr viel umfänglicher *regieren *lässt. Die biopolitische Produktivmachung der Körper beschränkt sich damit kaum auf klassische Einschließungsmilieus. Vielmehr ermöglichen Wearable-Technologien die Praktiken einer „Do-it-yourself-Überwachung“ (Bauman und Lyon 2013, S. 83), schaffen eine Umwelt partizipativer Services und personalisierter Nudges. Sie orchestrieren so Individuationsprozesse, ohne dabei die Register der Fernüberwachung, der panoptischen Erfassung und Zentralität (siehe „LumiHealth“ oder „Live Healthy SG“) gänzlich ad absurdum zu führen. In dieser Doppelbewegung erscheint es folgerichtig, dass die Regulierungen denen vormaliger Direktiven, d. h. den Interventionen in die „Fortpflanzung, die Geburten- und die Sterblichkeitsrate, das Gesundheitsniveau, die Lebensdauer, die Langlebigkeit mit allen ihren Variationsbedingungen“ (Foucault 2014, S. 69) stellenweise ähneln.^36^ Zudem ist im Rahmen der Wearable-basierten Selbstformung ein technologisch-experimenteller Möglichkeitshorizont maßgebend (im 18. Jahrhundert ging es derweil um statistische, mathematische Innovationen).

Zusammengefasst lassen sich vor dem Hintergrund der aktuellen, überwachungskapitalistisch grundierten Programme drei entscheidende Erweiterungen klassischer Biopolitik ausmachen: *Erstens* wird eine datenbasierte wie kybernetische Ausweitung der Analysemethoden, Regierungs- bzw. Regulierungstechniken kenntlich, da über die Zusammenführung von Verhaltens- und Vitaldaten nicht allein das Leben, sondern auch die *Lebensweisen* zum Kern der Erfassung und der Intervention werden. Dabei ergibt sich einerseits eine neoliberale, dezentrale Verantwortungsverlagerung eines „„aktivierenden Staat[es]“, der seine Bürger und Bürgerinnen aus der fürsorglichen Belagerung in die Freiheit der Selbstsorge entlässt und ihnen zumutet, ihre Lebensrisiken eigenverantwortlich zu managen“ (Bröckling 2004, S. 214). Andererseits geben nun, vermittelt qua Wearable, klarer, unmittelbarer und umfänglicher als je zuvor technologische Programmlogiken, ihre algorithmischen Bewertungs- und Wertsetzungssysteme an, wie sowohl individuell als auch kollektiv (für Krisen und andere Notfälle des Überlebens) vorzusorgen sei. Dabei fungieren die kybernetischen Feedbackmechanismen keineswegs unidirektional oder determinierend, um die „Produktionsmaschine“ Bevölkerung – die heute mit Bernard Stiegler (2009, S. 60) eher als „Konsumtionsmaschine“ zu begreifen ist – möglichst störungsfrei prozessieren zu lassen. Die Kontrollreflexe wirken subtiler, fokussieren eine anreizbasierte, personalisierte Selbstoptimierung wie die permanente Zirkulation von Kommunikation und Information.^37^ Man kann hier von einer angeleiteten Selbstkontrolle sprechen, einer Selbstverantwortung im Zeichen systematischer Selbstanpassung. In dieser Perspektive aktualisiert sich eine konstitutive Ambivalenz der, mit Foucault (2014, S. 69) formuliert, „biologischen, individualisierenden und spezifizierenden, auf Körperleistungen und Lebensprozesse bezogenen […] Technologie“: eine Verschränkung von Freiheit und Kontrolle, Individualisierung und Kollektivierung.

*Zweitens* lässt sich eine Vertiefung und Dynamisierung der Biopolitik qua algorithmischer Muster- bzw. Korrelationsanalyse nachzeichnen. In einer präemptiven Logik überschreibt die überwachungskapitalistische Formalisierung den vormals statistisch gefassten Gesellschaftskörper mit fluiden Datenpunkten und selbstlernenden Algorithmen, die seine Aktivitäten rückmelden, analysieren, als informatives Korrektiv wirken sollen. Dabei konzeptualisiert sie den Datenkörper als fraktale Masse aus „dividuell“ (Deleuze 1993, S. 258) gewordenen Subjekten, die sich als Knotenpunkte in dynamischen Datensätzen bzw. in ko-aktiven Feedbackschleifen ständiger Vergleichbarkeit bewegen. So konturiert sich eine ganz eigene, biopolitische Normierung, die sich aus den Mustern individueller Performanzen und der technologischen Verknüpfung der Körper selbst ergibt. Antoinette Rouvroy erkennt, wenngleich expliziter für den juridischen Kontext, in der sich hier abzeichnenden Dynamik die Grundierung einer „algorithmischen Gouvernementalität“. Diese richtet Subjekte indirekt wie informationslogisch aus, ohne dabei über diskursiv verhandelte gesellschaftliche Werte – die sich jenseits ökonomischer Effizienzlogik verdichten könnten – zu operieren:

algorithmic governmentality carefully avoids any direct confrontation with and impact on flesh and blood persons. […] What matters is the possibility of linking any trivial information or data left behind or voluntarily disclosed by individuals with other data gathered in heterogeneous contexts and to establish statistically meaningful correlations. The process bypasses individual consciousness and rationality […], and produces their „effects of government“ by anticipatively „adapting“ the informational and physical environment of persons according to what these persons are susceptible to do or wish to do, rather than by adapting persons to the norms which are dominant in a given environment. (Rouvroy 2013, S. 159)

Die expansive Erweiterung der körperlichen *Kennziffern* entfaltet *drittens* eine politökonomische Dynamik, die entlang einer klassisch solutionistischen Geisteshaltung verfährt (vgl. Morozov 2013), d. h. die Tendenz, jedes gesellschaftliche – nun auch: gesundheitliche – Problem über ein Mehr an Datenerhebung, ein Mehr an Sensoren, ein Mehr invasiver Technisierung „lösen“ zu wollen. Mit den technisch-innovativen Anwendungen und Schnittstellen zur *vitalen* Datenakkumulation formieren sich – wie an Apples und Googles Forschungsprojekten illustriert – neue Praxen der Kartierung, die ökonomische Interessen mit einer epistemischen Autorität verbinden. Die im Kern kybernetische Beschleunigung der Datafizierung und eine Spezialisierung auf kritische Schwellen wird vorangetrieben, um schließlich „digital biocapital“ (Lupton 2016, S. 117f.) zu erzeugen und so das ökonomisch grundierte Wissensregime zu erweitern. Kritisch zu betrachten ist nicht zuletzt die entstehende epistemische Asymmetrie – Zuboff (2018, S. 571) spricht in diesem Zusammenhang von den „Fähigkeiten des Überwachungskapitals, Unwissenheit in Wissen zu verwandeln“ –, die sich zu Gunsten jener Tech-Konzerne ausrichtet, die Einsicht in die proprietären Datenbestände des menschlichen Lebens haben und den Zugang zu diesem Wissen perspektivisch kontrollieren können.^38^

Mit Blick auf die jüngere Literatur zur Biopolitik wäre allerdings weniger zu fragen, ob die Regierung der Körper per se gut oder schlecht ist (vgl. Lorenzini 2021, S. 41; genereller Prozorov 2020). So lässt sich Foucaults Diagnose des biopolitischen Paradigmas durchaus nüchtern als Beschreibung einer Antwort auf ein Regierungsproblem lesen, das sich im 18. Jahrhundert verschärft stellte (vgl. ebd.). Im Anschluss an diese Perspektive ginge es vielmehr darum, zu überlegen, wie sich eine *demokratische Biopolitik* im Zeitalter der Digitalisierung ausgestalten könnte. Deutlich geworden ist mit Blick auf die ausgewählten Beispiele, dass gegenwärtige biopolitische Interventionen weniger repressiv als partizipativ durchgesetzt werden. Doch steht gerade die plattformökonomische Einbettung demokratischen Praxen biopolitischer Sorge – etwa die eigeninitiierte und gemeinschaftliche Förderung des Wissens über Krankheiten, die Entwicklung datengestützter Modi der Fürsorge^39^ – infrastrukturell entgegen, insofern sie unweigerlich epistemische Machtkonzentrationen in diesem Sektor fortschreibt. In einer solchen Perspektive stellt der Prozess dessen, was man eine *Überwachungskapitalisierung der Biopolitik* nennen kann, ein Problem dar, auf das demokratisch und pluralistisch zu antworten wäre. Die besprochenen Formen überwachungskapitalistischer Biopolitik können, wie im Anschluss zu zeigen sein wird, als Abgrenzungsfolie dienen. Die zu adressierenden demokratischen Defizite wurden zuletzt besonders im Zusammenhang mit der Corona-Pandemie deutlich und sollen abschließend konkretisiert und diskutiert werden.

## Infrastrukturelle Macht und vier Dimensionen einer digital-demokratischen Biopolitik

Im Zuge der geschilderten, multidimensionalen Expansion der Tech-Konzerne in den Gesundheitsmarkt überrascht schließlich wenig, dass Big Tech – besonders in seiner Rolle als Apologet einer überwachungskapitalistischen Biopolitik – die pandemische Ausnahmesituation als ein schockstrategisches Moment begreift, in dem sich die digitalen Zukünfte der Gesundheit genauer profilieren, Expertisen aufbauen und die smarten Erfassungslogiken verfeinern lassen. So entwickelte Google schnell und eigeninitiativ, während die Regierung Donald Trumps in den USA noch zögerte, das Problem des Virus überhaupt anzuerkennen, über das „Project Baseline“ bereits im März 2020 eine Website, die einzelne Bundesstaaten dabei unterstützte, Testverfahren zu koordinieren. In der Folge eröffnete man über 300 Teststationen, schuf ein zertifiziertes Test-Labor und für 16 US-Bundesstaaten die kostenlose Infrastruktur für effiziente Corona-Screenings,^40^ bei denen bis zum April 2021 über 3,9 Mio. Personen getestet wurden.^41^ Bereits im Herbst 2020 veröffentlichte „Project Baseline“ eine Reihe von Studien – von der Analyse der Immunreaktionen bis zur Erfassung von Antikörpern –, die u. a. Userdaten aus dem Testprogramm analysieren, um die wissenschaftliche Forschung zu Covid-19 „zu beschleunigen“ und die „nächste Generation von Gesundheitstools und Services zu entwickeln.“^42^ Wie diese dezidiert aussehen, lässt sich in dem von Verily entwickelten Service für betriebliches Gesundheitsmanagement „Healthy at Work“ erkennen, der Unternehmen und Institutionen Corona-Screenings anbietet: Neben kontinuierlichen Tests füllen Mitarbeitende hier täglich einen App-gestützten Symptomfragebogen aus, der sowohl den Arbeitgebern als auch Verily selbst zur Verfügung steht. Ferner bietet der Service digitale Werkzeuge für das Impfmanagement an und annonciert eine Kombination aus „population analytics and ongoing safety controls“^43^, die eine Wiederaufnahme der regulären Arbeit oder den sicheren Besuch der Universität ermöglichen sollen. So sinnvoll das Angebot erscheint – klar wird auch, dass hier ein erster wesentlicher Aspekt einer demokratischen Biopolitik prekär wird: **Privatheit bzw. informationelle Selbstbestimmung** treten leicht in den Hintergrund, da die individuelle Datensouveränität mit den programmatischen Sicherheitsdispositiven nicht selten in Konflikt gerät (vgl. Deutscher Ethikrat 2017).

Zusätzlich zu solchen eher stationären Services werden auch die smarten Devices der Konzerne zur Bekämpfung der Pandemie eingesetzt. So werden aktuell diverse wissenschaftliche Studien durchgeführt,^44^ die untersuchen, inwiefern etwa Wearables Coronavirusinfektionen noch vor spürbaren Symptomen – über die Abweichung der Verhaltens- bzw. Vitaldaten – erfassen können. Fitbit und die Apple Watch nehmen qua Infrastruktur in dieser Forschung Schlüsselpositionen ein, ermöglichen es Nutzer:innen, über tragbare Technologien und unterschiedliche Apps persönliche Daten zu spenden. Forscher:innen des Mount Sinai Health Systems haben etwa ermittelt, dass die Apple Watch Veränderungen in der Herzfrequenzvariabilität als mögliches Anzeichen einer Infektionen detektieren kann und bis zu sieben Tage vor merklichen Symptomen eine Infektion diagnostizieren könnte (vgl. Hirten et al. 2021).^45^ Das Scripps Research Institut^46^ oder die Universität Stanford haben ähnliche Ergebnisse publiziert, die weitere Verhaltensdaten (von der Aktivität bis zur Schrittzahl) und Biomarker (z. B. die Hauttemperatur) erfassten (vgl. Quer et al. 2021; Mishra et al. 2020). Derlei Studien erscheinen gerade in der pandemischen Ausnahmesituation hilfreich, werden zuweilen von den Tech-Konzernen selbst initiiert (siehe auch die „Apple Respiratory Study“^47^) und verschaffen den unternehmenseigenen Forschungen die angestrebte Legitimität, eröffnen nicht selten neue Märkte.

Grundlegend können diese Entwicklungen als Intensivierung der skizzierten Tendenzen einer bereits vor der Corona-Krise einsetzenden überwachungskapitalistischen Biopolitik – siehe „Apple Studies“ oder die Initiativen Singapurs – gelesen werden. Doch der pandemische Notstand hat nicht allein eine Ausweitung der Datenakkumulation und digitalen Infrastruktur zur Folge (vgl. hierzu auch Klein 2020). Er nuanciert auch, was Deleuze (1993, S. 161) treffend als „*Krankenhaus*-Regime“ beschrieb, das „potenzielle Kranke und Risiko-Gruppen erfaßt“. Genau in diesem Anwendungsfeld offenbaren sich aktuell signifikante Souveränitätsverschiebungen, die den Einfluss der Konzerne und die Macht ihrer technischen Systeme realpolitisch erfahrbar machen.

Dies wurde besonders in der Zusammenarbeit Apples und Googles beim Contact-Tracing im April 2020 kenntlich. Die Unternehmen erklärten, gemeinsam „an der Lösung eines der dringendsten Probleme der Welt“ bzw. an der Kontaktnachverfolgung via Smartphone zu arbeiten, die die Grundlage darstellen sollte, Infektionsketten schneller zu unterbrechen.^48^ Bereits im Juni präsentierten die Konzerne eine eigens entwickelte Schnittstelle, die einen dezentralen, anonymisierten Datenaustauch via Bluetooth möglich machte und heute den Standard für fast sämtliche, nationale Tracing-Apps in Europa bildet. Dass die beiden Konzerne so eine unumgängliche Norm schufen, angelaufene, staatliche wie paneuropäische Entwürfe und zentrale Lösungen (PEPP-PT etc.) ausschlossen, unterstrich den Ausnahmecharakter ihrer Autorität (vgl. Sharon 2020; Nosthoff und Maschewski 2021a; zur demokratietheoretischen Perspektivierung technologischer Infrastrukturen vgl. Berg und Staemmler 2020). So sehr man prinzipiell mit den dezentralen, datenschutzkonformen wie quelloffenen Lösungen wie der deutschen Corona-Warn-App zufrieden sein kann, so klar wurde auch, dass der öffentliche, kritische Diskurs (exemplarisch die Stellungnahmen vom Chaos Computer Club) eine eher untergeordnete Rolle spielte, schließlich „die Konzerne die Regeln dafür [diktieren], welche Daten überhaupt gespeichert und hochgeladen werden können und somit welche Art von Tracing-Technologie einzelne Staaten einsetzen“ (Dachwitz et al. 2021). Man kann hier eine Außerachtlassung formaldemokratischer Prozeduren oder die Durchsetzung einer biopolitischen Maßnahme qua Infrastruktur erkennen, die einem weiteren Aspekt *demokratischer Biopolitik* entgegensteht, da die **demokratische Deliberation** vernachlässigt, in gewisser Weise direkt-technokratisch überstimmt wurde.^49^

Dabei zeigt sich auch, dass überwachungskapitalistische Biopolitik nicht „inherently pluralistic“ ist, wie es Karsten Schubert zu Recht für eine demokratische Biopolitik einfordert: „Democratic biopolitics involves the participation of many actors – politicians, epidemiologists, and other experts, for example social scientists, and members of civil society, among others“ (Schubert 2020b). Die Tech-Monopolisten entziehen sich mit ihren „parastaatliche[n] Unternehmensstrukturen“ (Vogl 2021, S. 104) so nicht nur demokratischen Aushandlungsprozessen. Sie schaffen gleichsam Services und Tools, d. h. materielle Voraussetzungen, die eine ganz eigene, technologische Souveränität kennzeichnen; eine infrastrukturelle Macht,^50^ die weder demokratisch legitimiert noch **pluralistisch strukturiert** ist. Souverän ist so, wer die Standards setzt, den Informationsaustausch kanalisiert, kurz: wer über den Normalzustand entscheidet.

In dieser Hinsicht scheint auch der globale Notstand ein grundlegendes Merkmal des Überwachungskapitalismus zu prononcieren, d. h. mit Zuboff gesprochen: „die überwachungskapitalistische Herrschaft über die Wissensteilung in der Gesellschaft“ (2018, S. 571). Die informationsökonomische, ungleiche Distribution von datafizierten Einsichten erfährt mit Blick auf die Expansion in den Gesundheitsbereich dabei eine weitere Differenzierung. Denn die von Zuboff diagnostizierte epistemische Asymmetrie reduziert sich nicht nur – hierin scheint eine entscheidende Erweiterung der überwachungskapitalistischen Praxis zu bestehen – auf die Abschöpfung eines „Verhaltensüberschusses“. Vielmehr erweitern die Konzerne ihr *Angebot*, vermessen mit Ruhepuls und Blutsauerstoff in Echtzeit auch Vitalwerte und schaffen so proprietäre Datensätze, über die nur sie aggregiert verfügen. Mit Blick auf die immanenten, und gerade im Gesundheitssektor prävalenten, algorithmischen *biases* (vgl. Panch et al. 2019), die nicht selten strukturelle Benachteiligung prekarisierter Subjekte und minoritärer Gruppen reproduzieren,^51^ wird deutlich, dass die Ausweitung des Zugangs zu technologischen Analysetools nicht automatisch eine egalitäres Gefüge nach sich ziehen muss. Insgesamt ist damit ein vierter Punkt angesprochen, der einer demokratischen Biopolitik entgegensteht: So bedürfte es nicht nur einer Demokratisierung der Teilnahme, sondern auch der Teilhabe. Konkret hieße das, Einsicht in das generierte Wissen, in die Datenbanken von Leib und Leben und die Mechanismen ihrer Entwicklung (z. B. zu Forschungszwecken) einzufordern, sodass neben die Gleichheit im Sinne eines „equal access“ auch eine **epistemische Egalität **tritt und damit die Integration von Prinzipien wie Open Data und Open Source- bzw. Commonsmodellen (vgl. Stalder 2016, S. 245ff.; Ellerich-Groppe 2021).

Über ihre infrastrukturelle Macht und epistemischen Regime können die Tech-Konzerne aktuell fast exklusiv Tools und Services kreieren, die Körper immer genauer sondieren bzw. die Gesundheit kartieren und ihre Wirkmächtigkeit jenseits marktwirtschaftlicher Konkurrenz weiter entfalten. So wirken die Unternehmen nicht mehr als bloße Suchmaschinen oder Hardwarehersteller, sie erscheinen als enganliegende, vieldimensionale Infrastrukturen, die beständig diversifizieren. Dies tun sie kaum, um sich von alten Geschäftsfeldern zu *emanzipieren*, sondern um diese zu erweitern, zu vertiefen und immer nachhaltiger zu erfassen, wie bestimmte Lebensstile und ihre *Werte* korrelieren. Vor diesem Hintergrund scheint sich die beschriebene epistemische Asymmetrie von der bloßen Vorhersage und Instrumentalisierung des (Online‑)Verhaltens (vgl. Zuboff 2018) zu einer prädiktiven, biopolitischen Bestimmung dessen zu entwickeln (siehe neben „Project Baseline“ auch die Studien von Alphabets Tochterunternehmen DeepMind (vgl. Tomašev et al. 2019)), was überhaupt als normal, gesund oder ungesund zu gelten hat. Verbunden mit dieser Entwicklung sind wiederum ganz herkömmliche überwachungskapitalistische Topoi, d. h. algorithmische Empfehlungs- und Anreizsysteme, die gesunde Verhaltensweisen vorschlagen, Entscheidungen nahelegen bzw. vorstrukturieren. Damit bietet das Mehrwissen um die biopolitischen Risiken und Nebenwirkungen des Alltags schließlich auch neue ökonomische Chancen, neue Unternehmenszweige. Nur konsequent ist, dass Verily im Frühjahr 2021 in Kooperation mit dem Rückversicherer Swiss Re „Granular Insurance“ launchte, eine Stopp-Loss-Versicherung, die mit „data-driven insights“, „predictive analytics“^52^ und Wearables präzisere Risikokalkulationen ermöglichen soll.

Shoshana Zuboff macht für den Überwachungskapitalismus deutlich, dass die ungehinderte Akkumulation von Informationen eines „„dividuellen“ Kontrollmaterials“ (Deleuze) fast notwendigerweise zu einer Schwächung demokratischer Grundwerte führt: „Die Kombination von Wissen und Freiheit trägt zur Beschleunigung der Machtasymmetrie zwischen Überwachungskapitalisten und den Gesellschaften bei, in denen sie operieren.“ (Zuboff 2018, S. 571) Eine damit verbundene Einsicht ließe sich abschließend ebenso auf die Dimensionen einer überwachungskapitalistischen Biopolitik anwenden: „Unterbrechen“, warnt Zuboff (ebd.), „lässt sich dieser Kreislauf nur, wenn wir als Bürger, als Gesellschaft, ja als Zivilisation der Tatsache Rechnung tragen, dass Überwachungskapitalisten zu viel wissen, um in den Genuss der Freiheit zu kommen, die sie für sich beanspruchen.“

Tatsächlich hieße das: Es braucht politische Deliberation darüber, wie eine demokratische Biopolitik, nicht zuletzt auf infrastruktureller Ebene, ins Werk gesetzt, Partizipation im Gesundheitssektor ermöglicht werden kann, ohne dass plattformökonomische Machtkonzentrationen reproduziert werden. Adressiert werden müssten, so legt unsere Analyse nahe, vier Dimensionen, anhand deren sich eine Demokratisierung zu vollziehen hätte: *(1) Privatheit bzw. informationelle Selbstbestimmung, (2) demokratische Deliberation, (3) Pluralismus und (4) epistemische Egalität.*

## Footnotes

1 Der Begriff der „Individuierung“ deutet, im Gegensatz zur Individualisierung, eine eher prozessuale Emergenz von Subjektivität an, fokussiert das Individuum in einem nie abgeschlossenen Prozess seiner Entstehung. In einer Leibnizschen und Simondonschen Lesart setzt Deleuze das Individuum nicht als gegebene, abgeschlossene Einheit voraus, der Prozess der Individuierung lässt sich vielmehr als dynamisches Produkt einer Reihe von Modulationen begreifen. Im Fokus dieses relationalen Ansatzes steht also das Geschehen, in dem sich nur zeitweise stabile Einheiten herauskristallisieren. In den „techno-affektive[n] Gefügen“ (Schulz 2019) des Digitalen beschreibt Deleuze Subjektivitätsformierung so auch konsequent als einen Prozess, der auf ein „dividuum“ (lat. von das „Teilbare“) zielt: Subjektivität wird über digitale Codes binär beschreib- und teilbar, flexibilisier- und damit insbesondere im Kontrollregime des Plattformkapitalismus instrumentalisierbar.

2 Gerade die Covid-19-Pandemie zeigte ganz aktuell die Möglichkeiten der Telemedizin auf, während Tracking- und Tracingverfahren (via Smartphone oder Smartwatch) auch bei der Bekämpfung der Krise zum Einsatz kamen (vgl. Maschewski und Nosthoff 2021a).

3 Wir orientieren uns hierbei vornehmlich an Foucaults Biopolitik-Definition, wie sie in der *Der Wille zum Wissen* bestimmt wird, als „den Eintritt des Lebens und seiner Mechanismen in den Bereich der bewußten Kalküle und die Verwandlung des Macht-Wissens in einen Transformationsagenten des menschlichen Lebens“. Foucault beschreibt mit dem Begriff ein historisches Drehmoment im 18 Jahrhundert, bei dem sich „[z]um ersten Mal in der Geschichte […] das Biologische im Politischen [reflektiert]“ und markiert die „„biologische Modernitätsschwelle“ einer Gesellschaft […] dort, wo es in ihren politischen Strategien um die Existenz der Gattung selber geht“ (1977, S. 170f.).

4 Unter Big Tech werden hier die fünf US-amerikanischen Leitunternehmen der Technologiebranche begriffen, namentlich: Alphabet (Google), Amazon, Meta (Facebook), Apple und Microsoft.

5 Schon Michel Foucault erklärt, dass sich die Macht vor allem über Prozesse der „Akkumulation von Wissen, Beobachtungsmethoden, Aufzeichnungstechniken, Untersuchungs- und Forschungsverfahren und Verifikationsapparaten“ ausprägt und manifestiert (Foucault 1995, S. 49).

6 Während Cheney-Lippold unter dem Begriff der „soft biopolitics“ im Zuge algorithmischer Profilierung bereits 2011 einen internetbezogenen Modus dividueller Kontrolle bestimmt hat, fördert die Analyse körpernaher, tragbarer Technologien eine invasivere Form der Biopolitik zutage. Zur kontrollgesellschaftlichen Einordnung des Wearable-gestützten Selftrackings, vgl. Maschewski und Nosthoff (2021b).

7 Auch basisdemokratische Formen einer digitalen „Biopolitik von unten“ (Sotiris 2020) sind häufig von den zentral verwalteten Infrastrukturen der Plattformökonomie abhängig und verbildlichen eine problematische Dialektik von Freiheit und Kontrolle.

8 Zu Recht sind repressive Lesarten biopolitischer Intervention zuletzt aus unterschiedlichen Perspektiven kritisiert worden (vgl. Schubert 2020a). Aus einer dezidiert technologischen Perspektive hat Bratton eine „positive Biopolitik“ (2021) vorgeschlagen, die jedoch Gefahr läuft, durch eine ontologisierende Beschreibung einer Symbiose von Technologie und Leben konkrete Machtkonstellationen um Big-Tech zu naturalisieren und damit schließlich zu depolitisieren.

9 Eine konzise Ausnahme zur „Googlization of Health Research“ bildet die bereits 2018 erschienene Studie von Tamar Sharon, die allerdings jüngere Entwicklungen notwendigerweise unberücksichtigt lässt (vgl. Sharon 2018). Auch die einschlägigen Arbeiten zum „quantified Self“ oder „Lifeloggings“ (vgl. Selke 2014; Lupton 2016; Neff und Nafus 2016; Duttweiler et al. 2016; Bernard 2017) nehmen, zumal sie größtenteils vor den jüngst artikulierten Ambitionen und überwachungskapitalistischen Projektionen erschienen, zwar durchaus soziale Quantifizierungen im „metrischen Wir“ (Mau 2017) in den Blick. Doch gehen sie zumeist kaum dezidierter auf die Entwicklungen im Gesundheitsbereich ein.

10 Der Beitrag konzentriert sich auf die Konzerne Apple und Alphabet, aber auch Amazon forcierte in den letzten Jahren den Einstieg in den Gesundheitsmarkt. So stiegt der Online-Händler in den Markt für verschreibungspflichtige Medikamente („Amazon Pharmacy“) ein, entwickelte einen telemedizinischen Gesundheitsdienst („Amazon Care“) – zunächst nur für die eigenen Mitarbeiter:innen – und launchte 2020 mit „Amazon Halo“ auch ein Fitnesstracker (vgl. Maschewski und Nosthoff 2021c).

11 Siehe https://www.apple.com/de/watch/why-apple-watch/ (abgerufen am 28.10.2021).

12 Siehe https://www.apple.com/de/healthcare/ (abgerufen am 28.10.2021).

13 Vgl. https://www.apple.com/de/newsroom/2021/03/apple-hearing-study-shares-new-insights-on-hearing-health/ (abgerufen am 28.10.2021).

14 Vgl. https://www.apple.com/ios/research-app/ (abgerufen am 28.10.2021).

15 Ein weiteres Geschäftsmodell sind Gesundheitsmanagementprogramme in Unternehmen. Dabei werden Aktivitätswettbewerbe zwischen Abteilungen und Teams initiiert. Wenig überraschend ist dabei, dass die firmeneigene Fürsorge schnell auch die Dimension eines „exploited self-tracking“ (Lupton 2016, S. 115) annehmen kann (vgl. auch Mämecke 2016). Ein nennenswertes Beispiel ist Fitbits hauseigene Plattform „Care Engagement“, die trackinggestützte „Wellness-Programme“ für Arbeitnehmende anbietet (vgl. Charitsis 2019).

16 Vgl. https://www.attainbyaetna.com (abgerufen am 28.10.2021).

17 Darüber hinaus können die Versicherten ihre gesamten Gesundheitsdaten den beiden Unternehmen überantworten – für klinische Studien oder, wie es heißt, für Voraussagen über die Gesundheit der Bevölkerung (vgl. Shieber 2019).

18 Vgl. https://press.aboutamazon.com/news-releases/news-release-details/introducing-amazon-halo-and-amazon-halo-band-new-service-helps/ (abgerufen am 28.10.2021).

19 Siehe https://www.johnhancock.com/about-us/news/john-hancock/2020/08/amazon-and-john-hancock-announce-strategic-collaboration-aimed-at-helping-customers-improve-their-health-and-wellness.html (abgerufen am 28.10.2021).

20 Schaut man auf den deutschen Markt, so vollzieht sich auch wegen des gesetzlichen Solidaritätsprinzips die Entwicklung zögerlicher. Doch sowohl private als auch gesetzliche Anbieter (TK, AOK oder Barmer) offerieren bereits ähnliche Modelle für ein aktives Gesundheitsmanagement. Die private Generali Versicherung bietet so etwa seit 2016 ebenfalls ein Vitality-Programm an. vgl. https://www.generalivitality.com/de/de (abgerufen am 28.10.2021).

21 Niklas Ellerich-Groppe (2021) hat in seinem Beitrag in diesem Heft zudem Elemente einer Solidarisierung durch gängige Vermessungs- und Quantifizierungspraxen im Kontext des Self-Trackings herausgearbeitet und die damit verbundenen Herausforderungen (gerade hinsichtlich einer drohenden Entsolidarisierung) umfassend dargestellt.

22 Über die konzerneigenen Risikokapitalgesellschaften wie GV, Gradient Ventures oder CapitalG investiert Alphabet in unterschiedliche Start-ups im Gesundheitsbereich: 2020 z. B. 15 Mio. Dollar in das deutsch-amerikanische Unternehmen Klara, das Kommunikationsplattformen für Gesundheitsdienstleister entwickelt.

23 Siehe https://www.projectbaseline.com/ (abgerufen am 28.10.2021).

24 Siehe https://kstatic.googleusercontent.com/files/b0ebe863ce340ab878773f6af011f747b311dc8be7f2b9eb5d9a14926b199ef045d09eac3275540f8efdb39ebe8921b05cf7e89a27c8a0c1e117c7408eb0a644 (abgerufen am 28.10.2021).

25 Ebd.

26 Google verspricht, die Daten in einer „sicheren, verschlüsselten Database“ zu verwahren, doch die Geschäfts- und Teilnahmebedingungen offenbaren, dass die Informationen auch mit Drittparteien – etwa Pharmakonzernen und Geräteherstellern – zu kommerziellen oder Forschungszwecken geteilt werden.

27 Für die Zitate siehe Imagevideo auf der Webseite: https://www.projectbaseline.com/shape-healthtech/ (abgerufen am 28.10.2021).

28 Vgl. https://www.lumihealth.sg (abgerufen am 28.10.2021).

29 Siehe https://www.apple.com/de/newsroom/2020/09/singapore-and-apple-partner-on-national-health-initiative-using-apple-watch/ (abgerufen am 28.10.2021).

30 Siehe https://investor.fitbit.com/press-releases/press-release-details/2019/Fitbit-Collaborates-with-Singapores-Health-Promotion-Board-on-Population-Based-Public-Health-Initiative-in-Singapore/default.aspx (abgerufen am 28.10.2021).

31 Siehe https://www.lumihealth.sg (abgerufen am 28.10.2021).

32 Damit wäre auch Diagnosen widersprochen, die im Zuge der Ausweitung digitaler Technologien eine Kehrtwende von der Bio- zu einer ausschließlichen Psychopolitik erkennen, die es vermeintlich nur auf die „Psyche“ abgesehen habe (vgl. Han 2021, S. 9). Stattdessen kommt es zu einer Verknüpfung beider Macht- und Regierungsformen, *gerade *im kybernetischen Informationskapitalismus. Für den Hinweis auf Byung-Chul Hans *Infokratie* danken wir Sebastian Berg. Vgl. für eine differenzierte Analyse der im Informationskapitalismus zu Tage tretenden psychopolitischen Gouvernementalität Bösel (2021) in diesem Heft.

33 Genau dies ist immer wieder Gegenstand von Klagen: Im Juli 2020 verklagte etwa der Bund für Versicherte (BdV) die Generali-Versicherung wegen ihres Vitality-Programms. Der Vorstandsprecher Axel Kleinlein erklärt dazu: „Die Verbraucher erfahren zwar, dass gesundheitsrelevantes Verhalten den Beitrag verändern kann, nicht aber, an welchen Parametern dies bemessen wird.“ Und weiter: „An keiner Stelle des Vertragswerks sind hierfür klare und verständliche beziehungsweise genaue und nachvollziehbare Kriterien festgelegt.“ (zitiert nach: Hurtz 2020).

34 Zu den Begriffen der Norm, des Normalen und der Normalität im Kontext der Foucaultschen Überlegungen zur Biopolitik schreibt Thomas Lemke (2001, S. 113): „Die Sicherheitstechnologie repräsentiert das genaue Gegenteil des Disziplinarsystems: Geht dieses von einer (präskriptiven) Norm aus, so ist der Ausgangspunkt des Sicherheitssystems das (empirisch) Normale, das als Norm dient und weitere Differenzierungen erlaubt. Statt die Realität an einem zuvor definierten Soll auszurichten, nimmt die Sicherheitstechnologie die Realität selbst als Norm: als statistische Verteilung von Häufigkeiten, als Krankheits‑, Geburten- und Todesraten etc.“ Foucault (vgl. 2006, S. 87–132) selbst erläutert den Zusammenhang explizit in der dritten Vorlesung der *Geschichte der Gouvernementalität I *und spricht in diesem Kontext von einer „Ortung der Normalitätskurven“ (ebd.: 89) als einer gouvernementalen Praxis, die der disziplinaren Praxis der Durchsetzung einer Norm, die gleichzeitig das Abweichende markiert, eine entscheidende Variation hinzufügt.

35 Vgl. zu den epistemischen Dimensionen von Big Data in kritischer Perspektive Rieder und Simon (2017).

36 Überschneidungen ergeben sich insbesondere mit Blick auf Foucaults Analyse der Ökonomisierung und Produktivmachung des Subjekts im Zuge der Emergenz der Biopolitik im 18./19. Jahrhundert im Rahmen einer grundlegenden Liberalisierung des Staates und damit einhergehenden Kunst „der Menschenführung, die sich an der Bevölkerung als einer neuen politischen Figur orientiert“ (Lemke 2008, S. 80), wohingegen die mitunter totalitär-nationalistische Färbung einer „naturalistischen“ Biopolitik teils heterogene Zwecke verfolgt. Wir stützen uns hier ausschließlich auf Foucaults „relationalen“ Begriff der Biopolitik in Abgrenzung zu „naturalistischen“ oder „politizistischen“ Dimensionen. Zur Unterscheidung zwischen diesen Theorielinien vgl. Lemke 2008. Lemke (2008, S. 83) konkretisiert dazu, das „Problem“ einer „Analytik der Biopolitik“ im Sinne Foucaults sei „weder die Biologisierung der Politik noch die Politisierung der Biologie, da sie „Leben“ und „Politik“ als Elemente eines dynamischen Beziehungsgeflechts statt als äußerliche und unabhängige Größen begreift.“.

37 Insofern spielen in der Weise der Regulierung der überwachungskapitalistischen Biopolitik durchaus auch kybernetische Topoi eine Rolle, etwa die Vorstellung der Gesellschaft als homöostatisches System, das sich im Interesse des eigenen Überlebens permanent zur Selbstregulierung anleitet. Maria Muhles über Canguilhems Lebensbegriff erweiterte Lesart der Biopolitik Foucaults bietet in dieser Hinsicht einsichtsreiche Anknüpfungspunkte (vgl. Muhle 2013; insb. S. 205ff.).

38 Die Forschungsarbeiten sind in Teilen öffentlich verfügbar, so etwa die Studien Apples. Jedoch steht es den Tech-Konzernen frei, darüber zu entscheiden, mit welchen Institutionen sie welche Forschung durchführen, sodass sich die Forschung an bestimmte Bedingungen knüpft, die kaum mit Konzepten wie Open Data übereinstimmen.

39 Als ein Beispiel einer solchen demokratisch-digitalen Biopolitik kann Midata (siehe https://www.midata.coop abgerufen am 28.10.2021.) gelten. Als Gegenentwurf zu plattformökonomischen Modellen ist diese genossenschaftliche Plattform erwähnenswert, die Spenden von Gesundheitsdaten für unterschiedliche Institutionen ermöglicht und dabei neben Open Source-Standards die informationelle Selbstbestimmung ins Zentrum stellt (vgl. Blasimme et al. 2018). Die Corona-Warn-App wäre auf intentionaler Ebene ebenso ein Beispiel für eine Praxis datengestützter Fürsorge, doch wurden die Standards ihrer Entwicklung infrastrukturell von Apple und Google vorgegeben.

40 Teilnehmen konnten zunächst nur jene mit Google-Account. Wenig später bedurfte es eines Eignungstests, bei dem persönliche Gesundheitsdaten zur gegenwärtigen körperlichen Verfassung, zur Krankenhistorie und behandelnden Ärzt:innen wie auch der Wohnort an den Konzern überantwortet wurden.

41 Weitere Informationen unter: https://verily.com/2020-impact-report/.

42 Siehe https://www.projectbaseline.com/studies/covid-19/ (abgerufen am 28.10.2021).

43 Siehe https://verily.com/press/verily-launches-healthy-at-work-program-to-support-organizations-in-their-efforts-to-ensure-a-safe-return-to-work-and-school/ (abgerufen am 28.10.2021).

44 Diese Studien reichen von singulär privatwirtschaftlich durchgeführten Forschungen (siehe zu Fitbit: Natarajan et al. 2020) bis hin zu Public-private-Partnerships wie der Corona-Datenspende-App des Robert Koch Instituts, vgl. https://www.rki.de/DE/Content/InfAZ/N/Neuartiges_Coronavirus/Corona-Datenspende-allgemein.html (abgerufen am 28.10.2021).

45 Siehe https://www.mountsinai.org/about/newsroom/2021/mount-sinai-study-finds-wearable-devices-can-detect-covid19-symptoms-and-predict-diagnosis-pr (abgerufen am 28.10.2021).

46 Die „Detect“-Studie des Scripps Research Institute basiert auf einer Vorgängerstudie zur Erforschung von Grippesymptomatiken qua Fitbit, die bereits vor der Covid-19-Pandemie veröffentlicht wurde (vgl. Radin et al. 2020).

47 Vgl. https://clinicaltrials.gov/ct2/show/NCT04912180 (abgerufen am 28.10.2021).

48 Vgl. die Pressemitteilung beider Unternehmen: https://www.apple.com/de/newsroom/2020/04/apple-and-google-partner-on-covid-19-contact-tracing-technology (abgerufen am 28.10.2021). Zu den generellen ethischen Fragestellung rund um den Einsatz von Contact Tracing Apps vgl. Lucivero et al. (2020).

49 Vgl. generell zur Signifikanz der Deliberation und zur Bedeutung von Prozeduren der Aushandlung für eine Demokratisierung algorithmischer Prozesse Koster (2021) in diesem Heft.

50 Vgl. zur „infrastrukturellen Macht“ auch ihre Definition und Bedeutung im Feld der sozialen Plattformen (Maschewski und Nosthoff 2021d).

51 Ein Beispiel dafür sind bildgebende Verfahren wie etwa die App Alphabets zur Erkennung von Hautkrankheiten, die weitaus besser bei weißen Anwender:innen funktioniert als bei People of Color. (Vgl. Wieschollek 2021).

52 Siehe https://granularinsurance.com (abgerufen am 28.10.2021).
